# Truncated and modified amyloid-beta species

**DOI:** 10.1186/alzrt258

**Published:** 2014-05-26

**Authors:** Markus P Kummer, Michael T Heneka

**Affiliations:** 1 Department of Neurology, University Hospital Bonn, Sigmund-Freud-Strasse 25, 53127 Bonn, Germany; 2 German Center for Neurodegenerative Diseases (DZNE), Holbeinstrasse 15, 53117 Bonn, Germany

## Abstract

Alzheimer’s disease pathology is closely connected to the processing of the amyloid precursor protein (APP) resulting in the formation of a variety of amyloid-beta (Aβ) peptides. They are found as insoluble aggregates in senile plaques, the histopathological hallmark of the disease. These peptides are also found in soluble, mostly monomeric and dimeric, forms in the interstitial and cerebrospinal fluid. Due to the combination of several enzymatic activities during APP processing, Aβ peptides exist in multiple isoforms possessing different N-termini and C-termini. These peptides include, to a certain extent, part of the juxtamembrane and transmembrane domain of APP. Besides differences in size, post-translational modifications of Aβ – including oxidation, phosphorylation, nitration, racemization, isomerization, pyroglutamylation, and glycosylation – generate a plethora of peptides with different physiological and pathological properties that may modulate disease progression.

## Introduction

Since its identification from senile plaques, amyloid-beta (Aβ) peptide has been considered to play a central role in the pathology of Alzheimer’s disease (AD) [1]. Aβ is thought to accumulate in AD cases because of an imbalance in the production and clearance of this peptide resulting in the formation of the characteristic amyloid plaques in specific brain regions. The large majority of AD cases are of sporadic nature, showing inefficient removal of Aβ [2], whereas a minority of cases is caused by genetic mutations (familial AD) with an onset typically below age 65 years. Most of these cases are caused by autosomal dominant mutations in genes related to the processing of amyloid precursor protein (APP) leading to increased production of Aβ.

**Figure 1:**
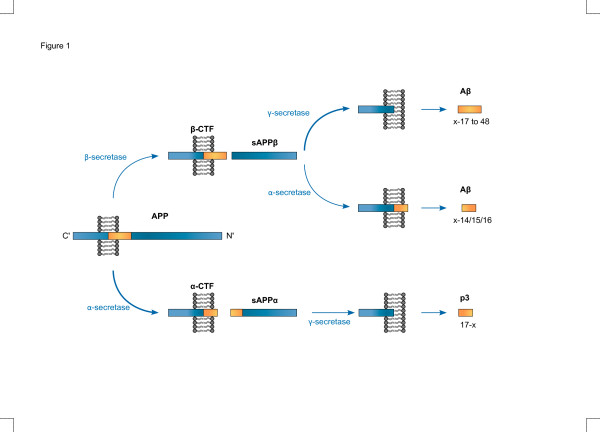
**Generation of different amyloid-beta domain-derived peptides from the amyloid precursor protein.** The amyloid precursor protein (APP) is preferentially cleaved in the non-amyloidogenic pathway by α-secretase into a large ectodomain called sAPPα, and into a C-terminal stub called α-C-terminal fragment (α-CTF), which is further processed by γ-secretase into p3 peptides. Alternatively, APP may be cleaved in the amyloidogenic pathway by β-secretase into an ectodomain called sAPPβ and into a longer C-terminal stub called β-C-terminal fragment (β-CTF). This stub is preferentially cleaved by γ-secretase to generate amyloid-beta (Aβ) peptides, but some β-CTF precursors are cleaved by α-secretase resulting in C-terminal truncated Aβ species.

To generate Aβ from APP, the precursor has to be cleaved by a set of two proteases (Figure 1). The first cleavage occurs at a luminal, juxtamembrane position, resulting in the formation of a membrane-bound C-terminal stub that, in a subsequent step, is cleaved by an unconventional protease complex, called γ-secretase, within the transmembrane domain, thereby liberating Aβ. Both cleavages have been shown to be imprecise. β-site amyloid precursor protein cleaving enzyme 1 (BACE1), the enzyme that conducts the initial processing, step-cleaves APP at a minimum of two positions, whereas γ-secretase generates a variety of different Aβs spanning 34 to 50 amino acids in length.

**Figure 2:**
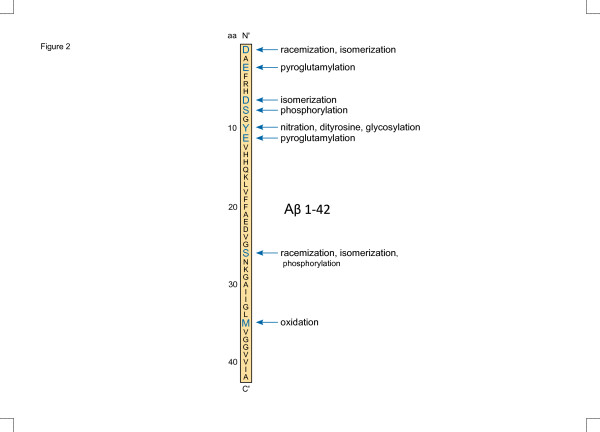
**Positions of post-translational modifications in amyloid-beta 1–42.** Blue letters indicate amino acid residues that are subject to post-translational modifications (PTM). Multiple PTM have been observed for some amino acid (aa) residues. Aβ, amyloid-beta.

Most AD cases are sporadic, however, without changes in the production of the Aβ peptide. The propensity to form aggregates and toxic species may therefore be driven by factors other than changes in the production of certain Aβ peptides. Several post-translational modifications (PTMs) have been discovered that on the whole increase the aggregation rate of Aβ (Figure 2). Some of these modifications, such as oxidation and nitration, are obviously induced by the inflammatory milieu that is a component of AD [3].

PTMs can function as a molecular switch to evoke cellular responses, but one should consider that they may also be a result of protein aging that is random and without any physiological impact.

## Truncated amyloid-beta species

### Amyloid-beta species generated by α-secretase, β-secretase, and γ-secretase

Three enzymatic activities are involved in APP processing, and were named α-secretase, β-secretase, and γ-secretase at a time when their molecular identities were unknown. APP exists in several isoforms ranging from 695 to 770 amino acids in length, including the domain from which the Aβ peptide derives. In APP695 (the most abundant isoform in the brain) this domain ranges from amino acids 597 to 638. In an initial step, APP is cleaved at a juxtamembrane position at the luminal side of the membrane. This cleavage is mediated at different positions by either α-secretase or β-secretase in different compartments of the cell [4]. The majority of APP molecules in non-neuronal cells are initially cleaved by α-secretase between positions 16 and 17 of the Aβ domain. This is the so-called non-amyloidogenic pathway since the cleavage occurs within the Aβ domain, thereby preventing the production of Aβ. This event generates a stub called α-C-terminal fragment as well as a large ectodomain called sAPPα. Several members of the ADAM family of proteases are able to mediate this cleavage, but in neurons this function is likely to be exerted by the constitutively active ADAM10 protease [5].

In the amyloidogenic pathway, leading to the production of Aβ peptides, β-secretase mediates the initial rate-limiting step. The membrane-bound aspartyl-protease BACE1 has been identified as the responsible enzyme. APP is cleaved by this enzyme before position 1 of the Aβ domain [6], resulting in the release of a large ectodomain and the formation of a stub called β-C-terminal fragment. In addition, BACE1 can also cleave APP within the Aβ domain between positions 10 and 11 (β′ site) [7]. Subsequently, both N-terminally cleaved precursors are further processed by γ-secretase, a complex that consists of at least the proteins APH-1, PEN-2, nicastrin and presenilin 1 or presenilin 2 [8]. The transmembrane proteins presenilin 1 and presenilin 2 possess two critical aspartyl residues that are part of the catalytic domain of this γ-secretase subunit. The cleavage occurs within the transmembrane domain of APP, generating C-terminally truncated peptides ending with amino acids 37 to 43, due to an imprecise cleavage of these enzymes. The resulting peptides are liberated into extracellular fluids such as cerebrospinal fluid (CSF), plasma or interstitial fluid. This phenomenon is not fully understood, but endoproteolysis is thought to occur stepwise, cleaving the C-terminal stubs several times within their transmembrane domain. These cleavages are approximately three amino acids apart [9,10]: one at amino acid 48 or 49, followed by another one at position 45 or 46, and ending with a final cleavage most often at position 38, 40 or 42. At least in the CSF of nondemented controls, about one-half of the Aβ ends at amino acid 40, 16% ends at amino acid 38, and 10% ends at amino acid 42 [11]. Aβ species ending with the alanine at position 42 have a stronger tendency to aggregate as compared with Aβ1–40. These species are thought to be the driving factor for the formation of amyloid plaques and the neurotoxic effects [12]. During this last step, other minor cleavage sites have been observed at positions 34, 37, 39, and 43 [9]. There are even shorter Aβ isoforms (Aβ1–17/18/19/20) that depend on γ-secretase [13,14] but the precise mechanism of their generation is unknown.

Both processing pathways lead to a large variety of peptides that start either at position 1 or position 11 caused by cleavage by BACE1 or at position 17 mediated by α-secretase. The latter peptides are called p3 [15] but are not found in senile plaques, and neither do they yet appear to have any pathological or physiological role. Most of these N-terminal starting points are found in combination with the heterogeneity caused by the cleavage of γ-secretase.

To make things even more complicated, the amyloidogenic pathway and the nonamyloidogenic pathway seem not to be mutually exclusive. There are shorter isoforms of Aβ (Aβ1–14/15/16) present in the CSF that do not depend on γ-secretase cleavage but are sensitive to inhibitors of α-secretase. Interestingly, Aβ1–14/15/16 increase after γ-secretase treatment in CSF [16,17]. This leads to the conclusion that C-terminal BACE1-cleaved stubs (C99) are not exclusive substrates for γ-secretase, and that C99 can reach compartments with α-secretase activity resulting in the liberation of Aβ1–14/15/16 along alternative pathways [13,14,18].

### N-terminal amyloid-beta truncations independent of α-secretase or β-secretase

There are several N-terminal truncations observed in AD that cannot be explained by the action of the above-described enzymes [19]. In general, N-terminal truncations make up the majority of Aβ species in AD [20,21] but not in the transgenic mice mouse model, which might explain the differences in the molecular mechanisms of amyloid deposition [20,22]. In addition, the shortening of the N-terminus increases the propensity of Aβ to form aggregates *in vitro*[23]. Since Aβ is degraded by several secretory proteases, such as insulin-degrading enzyme and neprilysin among others [24], it is possible that truncations arise from these enzymes.

The 2-x Aβ species has been found to be increased in the brains of AD patients [25,26] and decreased in the CSF of AD patients [27]. There was a suggestion that this species might derive from the combined action of BACE1 followed by aminopeptidase A [25]. Recently, the metalloprotease meprin-beta was reported to initially shed APP in a BACE1-independent fashion, releasing different Aβ species with several cleavage sites. These sites are reported to be identical with or proximal to the known β-secretase cleavage site [28], and overexpression of meprin-beta generates Aβ2–40 [11,25]. However, further studies and appropriate mouse models are necessary to investigate the contribution of meprin-beta in AD.

The 3-x Aβ species has been detected in a mouse model of AD [20,29,30] and in senile plaques from brains of late AD cases [31]. It has been suggested that this species is generated by Cu^2+^-mediated amide hydrolysis or the peptide bond between amino acids 2 and 3 of Aβ [32].

One of the first Aβ peptides reported was the N-terminal truncated 4-x species [33]. In comparison with other species, Aβ4–42 was found to be relatively abundant in AD and vascular dementia [34]. *In vivo*, mice overexpressing Aβ4–42 suffer from a massive CA1 pyramidal neuronal loss, accompanied by memory dysfunction [35]. There is so far no candidate enzyme that mediates this cleavage.

The 5-x Aβ species was initially described in cells expressing an APP lacking the C-terminal 31 amino acids, but has also been discovered in AD patients using a 5-x Aβ neo-epitope antibody [36,37] and in nondemented controls by mass spectometry [21]. Interestingly, using APP-overexpressing cell lines, inhibition of BACE1 resulted in the appearance of Aβ5–40 [38,39]. This species has also been detected in the 5XFAD mouse model of AD [29] and in the CSF of dogs treated with BACE1 inhibitor [39].

The largest amino truncations, aside from that at position 11 mediated by BACE1, are so far the cleavages that occur before amino acids 7, 8 and 9 observed in the brains of AD patients [21,22]. A candidate enzyme for the formation of the 8-x Aβ species might be angiotensin-converting enzyme [40], but so far there are no *in vivo* data supporting this pathway.

## Amyloid-beta species modified by post-translational modification

### Oxidation

The most prominent site of oxidative changes within Aβ is the methionine at position 35 (Met35). Increased oxidative stress has been described in the brains of mild cognitive impaired and AD patients. Part of this oxidative stress is mediated by the Aβ peptide itself, but other mechanisms, such as inflammatory inducers and others, may also be relevant.

Oxidation of Met35 to methionine sulfoxide in AD was first observed years ago [41]. The reaction proceeds through a radical intermediate that can be prevented by the use of radical scavengers [42]. Several studies demonstrated that oxidation of Met35 impedes the formation of Aβ protofibrils and fibrils from monomers [43,44]. A role for Met35-oxidized Aβ in the formation of ion-channel-like structures in lipid membranes has also been reported [45].

### Phosphorylation

In theory, Aβ possesses three potential phosphorylation sites at serine residues 8 and 26 and at tyrosine residue 10. There are numerous examples of phosphorylated extracellular/luminal protein suggesting the existence of extracellular kinases that facilitate this PTM. Phosphorylation of the serine at position 26 has been described in NT2 neurons and AD brains [46]. *In vitro*, this PTM is generated by the action of the cdc2 kinase. In turn, using a cdc2 kinase inhibitor, the neurotoxic effect of Aβ on NT-2 neurons can be reduced [46].

Phosphorylation of Aβ at serine 8 has been studied in more detail. Using phospho-serine-8-specific Aβ antibodies revealed the presence of phosphorylated Aβ in AD mouse models and AD. Under pathological conditions this species was found to be localized to amyloid plaques [47], but could also be found intracellularly [48]. Biophysically, this PTM increases the formation of oligomeric Aβ aggregates that represent nuclei for fibrillization. This species shows increased toxicity in drosophila models as compared with nonphosphorylated Aβ [47]. In addition, serine 8-phosphorylated Aβ is resistant to degradation by insulin degrading enzyme [49].

### Nitric-oxide-caused modifications

Nitric oxide (NO) induces several PTMs, including the formation of S-nitrothiols at cysteine residues and nitration and dityrosine formation at tyrosine residues [50]. Increased presence of these NO-caused PTMs has been observed in AD [51,52]. The source of NO during AD is most probably the enzyme NOS2, which is upregulated in AD [53,54]. As a molecular target, tyrosine 10 of Aβ has been shown to increase the propensity of Aβ to aggregate and has been identified in the core of the amyloid plaques [55]. The reaction of Aβ with peroxynitrite, an intermediate NO product, *in vitro* has been shown to generate both nitrated Aβ and dityrosine-coupled Aβ. The latter modification could also be detected in the core of amyloid plaques [55] and may stabilize Aβ dimers [56]. Nitrated Aβ was able to initiate plaque formation in APP/PS1 mice, suggesting a central role during the early phase of AD [55]. Hippocampal long-term potentiation was suppressed more by nitrated Aβ compared with non-nitrated Aβ. This demonstrates that this PTM is involved in both the functional and structural changes in AD. In addition, formation of this Aβ species is favored by oxidative stress [56,57].

### Glycosylations

Mass spectrometry analysis of controls and AD patients revealed the presence of O-glycosylated Aβ species in CSF [58]. The glycoforms included monosialylated, disialylated, and trisialylated modifications, as well as lactone modifications. The exact molecular nature of the glycosylation has not been determined and could therefore be GlcNAc, GalNAc, or ManNAc in either α-linkage or β-linkage to the conjugated amino acid. Glycosylation occurred on Aβ1–15/16/17/18/19/20, Aβ3–15, Aβ4–15, Aβ4–17, and Aβ5–17 peptides, with Aβ1–15 and Aβ1–17 peptides being the most abundant of all Aβ1–X glycopeptides. The absolute concentration for glycosylated Aβ1–15 was calculated to be 10 to 30 pg/ml CSF, whereas that for unglycosylated Aβ1–15 ranged from 100 to 200 pg/ml. For Aβ1–15 and Aβ1–17 the glycosylations were selectively attached to tyrosine 10 of the Aβ sequence. The lack of glycosylated Aβ1-40/42 peptides in CSF led to the conclusion that tyrosine 10 O-glycosylation in APP modifies the γ-secretase cleavage, because of the proximity of this glycosylation to the transmembrane domain [58].

### Pyroglutamylation

The initial attempts to identity the N-terminus of Aβ revealed a minor species beginning with glutamic acid at position 3 [59]. Development of specific antibodies to pyroglutamate Aβ demonstrated its weak solubility and presence in amyloid plaques [60]. As an initial step, formation of pyroglutamate-modified Aβ at position 3 (3pE-Aβ) requires the removal of the first two amino acids from Aβ. Aminopeptidase A has been suggested as an enzyme facilitating this processing [61], but this has yet to be proven. In addition, spontaneous amide hydrolysis by Cu^2+^ has been reported [32]. Further, another pyroglutamate modification at aspartate 11 was discovered (11pE-Aβ) [41,62]. This species may arise from the alternative BACE1 cleavage side in APP [63,64]. In a subsequent step, the terminal glutamate is converted to a pyroglutamate in a dehydration reaction. This reaction can be catalyzed by the enzyme glutaminyl cyclase [65], which is increased in AD [66]. Reduction of glutaminyl cyclase results in reduced formation of pyroglutamate Aβ *in vitro*[67] and *in vivo*[66,68]. Further, reduced glutaminyl cyclase expression in AD mouse models is accompanied by reductions in Aβ40/42 levels, reduced plaque burden, inflammatory reaction, and improved memory and spatial learning [66,68].

*In vitro*, 3pE-Aβ42 has a similar toxicological profile on neuronal cells to that of Aβ1–42 [69], which was confirmed by intracerebroventricular injections of either 3pE-Aβ42 or Aβ1–42 [70]. Like many changes in the N-terminus of Aβ, 3pE-Aβ and 11pE-Aβ show increased propensity to aggregate and to form β-sheets *in vitro*. This may be caused by higher hydrophobicity since two charges are lost during conversion [71]. pE-Aβ has been detected in a variety of AD mouse models, yet the time of first appearance during pathology varies strongly between different mouse models – ranging from 2 months in the APP/PS1KI model [72], to 16 months in the Tg2576 model [73], to 15 months in the APP23 model [20]. Interestingly, there has been extensive neurotoxicity described in mouse models that generate pyroglutamate-modified Aβ [74,75].

### Isomerization

Peptides are susceptible to spontaneous, non-enzymatic isomerization particularly at asparagine and aspartate residues, resulting in the formation of isoaspartate. These aspartyl-bond isomerizations affect the secondary structure of the peptide and may therefore be critical for the development of pathological processes such as aggregation and deposition [76]. In parenchymal plaque core preparations, the predominant species of Aβ at the aspartyl residues 1 and 7 is the l-isoaspartyl form [77]. Interestingly, the amount of isoaspartyl residues in Aβ preparations from vascular depositions is lower compared with preparations from senile plaques [77,78], suggesting that Aβ from plaques are older since this PTM increases over the lifetime. Isomerization of aspartate 23 has not so far been detected by biochemical means in AD brains.

*In vitro*, substitutions of positions 1, 7 and 23 of Aβ by isoaspartate increased the tendency of these peptides to form β-pleated sheets [78], to form aggregates [79,80] and to contribute to the enhanced insolubility and resistance to enzymatic degradation [81]. The presence of isoaspartate-7 Aβ detected by specific antibodies was suggested to be an indicator of plaque age since this was found mostly in the core of amyloid plaques and correlated with dementia severity [82].

### Racemization

Racemization is the process of conversion of enantiomers so that both enantiomers are present. In the case of amino acids this is the conversion from the l-form to the d-form, especially at seryl and aspartyl residues. Presence of d-enantiomers of aspartyl and seryl residues in Aβ have long been described [83-85]. As for isomerized Aβ, the presence of racemized aspartyl residues in Aβ was found to be higher in amyloid plaques compared with vascular Aβ [77]. *In vitro*, racemization of Aβ can be induced by radicals [86]. In a recent study, the enrichment of d-Asp^1^ as well as of its isomer d-isoAsp^1^ could be demonstrated in the tissue of AD patients by mass spectometry [87]. d-Ser^26^-Aβ1–40 possesses a stronger tendency to form fibrils [84].

## Use of amyloid-beta species as diagnostic marker and as a target for treatment

Because of its disease-dependent regulation in the CSF, Aβ has been used as a biomarker for AD. In particular, the concentration of Aβ1–42 in the CSF undergoes a characteristic drop during disease progression, which is interpreted as the consequence of the ongoing parenchymal Aβ deposition in senile plaques. In addition, the CSF levels of Aβ1–40 remain constant so that the ratio of Aβ1–42/Aβ1–40 is a valuable predictor. Since the C-terminal truncated Aβ1–14/15 species are elevated after inhibition of γ-secretase, their use as a readout for trials aiming at the modulation of γ-secretase has been suggested [88]. The sensitivity can be increased by combining the markers Aβ1–42, Aβ1–38 and pTau, resulting in a 94% discrimination between nondemented patients and AD patients [89]. Beyond the diagnosis of AD, the concentration of Aβ1–38 is specifically lowered in the CSF of patients affected by fronto-temperal dementia [90].

The search for human auto-antibodies in plasma and CSF revealed that most antibodies recognized either oligomeric or post-translationally modified Aβ [91], suggesting that this species might be a relevant target for passive or active immunotherapy. Since it is unknown whether unmodified Aβ has a physiological role, there is a certain risk that preventive immunotherapy may cause undesirable effects. In addition, decreased levels of Aβ autoantibodies might even contribute to disease progression as has been shown for pyroglutamate-modified Aβ [92]. The immunotherapy directed at pathological Aβ species is therefore a road that should be followed.

Several PTMs of Aβ increase the tendency of Aβ to aggregate [46,57,91] and are found in the core of amyloid plaques [55,93], suggesting that plaque formation depends on them. The use of inhibitors that interfere with their formation therefore has to be evaluated. In particular, inhibitors for glutaminyl cyclase [66], the relevant kinases for the phosphorylation of Aβ and NO-producing enzymes, especially NOS2 [55], should be tested as a therapeutic option.

## Conclusion

From the plethora of Aβ species, some are generated very early during APP processing, some are modified immediately afterwards, or others are found or generated in certain cellular or extracellular compartments, and some are actually markers for the slow to non-existent turnover of these peptides in amyloid plaques. Certain regions of Aβ obviously contribute differentially to its properties, like N-terminal truncations, and certain amino acids are hotspots for PTMs. There is a possibility that some of these species might serve as excellent diagnostic markers or therapeutic targets in the future.

## Abbreviations

AD: Alzheimer’s disease; APP: Amyloid precursor protein; Aβ: Amyloid-beta; BACE1: β-site amyloid precursor protein cleaving enzyme 1; CSF: Cerebrospinal fluid; Met35: Methionine at position 35; NO: Nitric oxide; PTM: Post-translational modification.

## Competing interests

The authors declare that they applied for a patent for the use of nitrated Aβ.

## Authors’ contributions

MPK and MTH wrote the manuscript. Both authors read and approved the final manuscript.
